# Reliability and validity of the Chinese version of the self-directed learning instrument in Chinese nursing students

**DOI:** 10.1186/s12912-023-01201-3

**Published:** 2023-02-24

**Authors:** Ziyun Gao, Lin Zhang, Jianing Ma, Hong Sun, Mengya Hu, Meiding Wang, Haiyang Liu, Leilei Guo

**Affiliations:** 1 School of Nursing, Jinzhou Medical University, Linghe District Jinzhou City, No.40, Section 3, Songpo Road, Liaoning Province Jinzhou, People’s Republic of China; 2 Department of Internal Medicine Nursing, School of Nursing, Wannan Medical College, 22 Wenchang West Road, Higher Education Park, An Hui Province Wuhu City, People’s Republic of China; 3 Student Health Center, Wannan Medical College, 22 Wenchang West Road, Higher Education Park, An Hui Province Wuhu City, People’s Republic of China

**Keywords:** Self-directed learning, Learning styles, Factor analysis, Nursing students, Reliability, Validity

## Abstract

**Background:**

In a rapidly changing healthcare environment, Self-directed learning (SDL) ability is recognized as a crucial condition for nursing students and nurse to deal with severe challenges positively. Developing SDL ability is becoming more and more important among nursing students. SDL is related to nursing students enhancing their own knowledge, skills and maintaining lifelong learning. This study is aim at translating the Self-directed Learning Instrument (SDLI) into Chinese and verify its reliability and validity among nursing students.

**Methods:**

The study adopted a cross-sectional design and the multistage sampling design. The SDLI was translated into Chinese, and the reliability and validity of the scale were tested among 975 nursing students.

**Results:**

The Cronbach’s α value of the Chinese version of SDLI was 0.916. The split-half reliability coefficient was 0.829, and the retest coefficient was 0.884. The content validity index of the scale was 0.95. Furthermore, the four-factors model was obtained by using exploratory factor analysis, explaining 55.418% variance, and the communalities of the items ranged from 0.401 to 0.664. With modified confirmatory factor analysis, the fit indices were chi-square/degree of freedom (CMIN/DF) = 2.285, the comparative fit index (CFI) = 0.947, and the tucker lewis index (TLI) was 0.938. And, the model fitting indexes were all in the acceptable range and confirmatory factor analysis indicated that the model fit the SDLI well.

**Conclusion:**

The Chinese version of SDLI has good validity and reliability among nursing students. It can be used to measure the SDL ability of nursing students in China.

**Supplementary Information:**

The online version contains supplementary material available at 10.1186/s12912-023-01201-3.

## Background

Nowadays, the medical environment is developing rapidly, the medical demand is increasing, the time to update medical knowledge is getting shorter and shorter, and the medical technology is constantly improving, which requires nurses to constantly update their professional ability to provide effective and high-quality care [1–3]. In the wake of the novel Coronavirus outbreak in 2019, clinical practice at home and abroad has been suspended and postponed, offline courses and experimental operation courses have also been disrupted. Delayed start of school nationwide, extended vacation time, students staying home for long periods of time and unable to attend school regularly. The independent learning ability among nursing students has been challenged. so it imperative for nursing students to rely on SDL to access information, learn knowledge and develop skills [4]. It is of great significance for medical students to master medical knowledge and be competent in clinical work to use their own time effectively [5, 6]. All of these require that nurses continue to improve their knowledge through the Self-directed learning (SDL) to adapt to the dynamic clinical environment [7–9].

SDL is a process. In this process, with or without the help of others, learners set goals and plans based on their learning needs, identify human and material resources, select and implement appropriate strategies, and assess the level of learning achieved [10].

SDL is more effective than traditional teaching in improving students' skills, knowledge and attitudes [11–15]. Nurses with SDL ability can help them to master relevant professional knowledge in a more timely and effective manner and provide high-quality nursing services for patients. Once nursing students have acquired SDL during their undergraduate nursing years, they will continue to keep this learning way so after graduation to develop their nursing knowledge and skills [16–19]. More importantly, SDL is an essential attribute of lifelong learning [20–22]. SDL enables students not only to acquire personal responsibility and learning mastery through independent learning, and to possess the basic qualities of being a nurse, such as thoughtfulness, responsibility and self-confidence [23, 24], but also to become independent, capable learners of skills and lifelong learners [23, 25]. Therefore, in order to help nursing students become qualified nurses, it is very crucial to develop SDL ability of nursing students.

With the increasing concern of SDL ability among nurses and other health care workers [26], it was important to find suitable tools to evaluate the SDL ability among nursing students. The scales commonly used to evaluate nursing students' self-directed ability are, SDLRS [27], SRSSDL [28–30], SDLRSNE [31–33], SDLI [34]. These scales are based on Knowles’s theory and measure the SDL ability of nursing students. Items range from 20 to 60 in these scales. SDLI is a shorter tool with only 20 items [34]. A simple tool can improve participants' willingness and accuracy to complete the questionnaire. In addition, most of these scales are developed in Western countries, and the relevant content of measurement is different. Some of the content is not suitable for Asian countries and lacks psychometric characteristics. Based on professor Lucia Cadorin's research, each assessment tool took into account a number of psychometric characteristics, but the quality of the methods used was generally poor, except for SDLI. Therefore, SDLI can be recommended to assess SDL competence of nursing students [7]. Taiwanese scholar Su-fen Cheng developed the SDLI scale in 2010 by combining SDL ability with nursing. SDLI consists of 4 dimensions and 20 items. It is a simple and effective assessment tool. SDLI assesses the following aspects: students' learning motivation, planning and implementation, self-monitoring and interpersonal communication. SDLI conducted a comprehensive and effective assessment of the SDL ability of nurses. And, the scale showed good reliability, validity and accuracy in Taiwan population, and could be used to measure the SDL ability of nursing students. Moreover there are also language differences between traditional Chinese and simplified Chinese, which may produce inaccurate results [35]. The traditional Chinese version of SDLI is not suitable for use in mainland Chinese environment. In view of these problems, it is important to evaluate the cultural and linguistic equivalence of translated scales before using them in mainland Chinese contexts. This study aims to verify the validity and reliability of the Chinese version of SDLI among nursing students.

## Methods

### Design and sample

The study adopted a cross-sectional design and the multistage sampling design. From June to December 2015, nursing students from several medical colleges in Jinzhou city and Dalian City, Liaoning Province, China were surveyed. The investigators were mainly nursing graduate students who participated in the study. They had received uniform training on how to use standardized language and instructions. All participants completed the tests voluntarily. Approval for the study was obtained from the College of Nursing’s Research Committee at Jinzhou Medical University (2,015,007). Inclusion criteria: Full-time nursing students; Informed consent and voluntary participation in this study; Nursing students who are able to speak or read and communicate easily with investigators. Exclusion criteria: Unwilling to participate in this study. Based on the criterion proposed by Kendall that requires a mini-mum of 10 respondents per item [36]. And with the purpose of ensuring the precision of the results of this study, a total of 975 nursing students were surveyed by questionnaire.

### The instrument

The SDLI [34] is a 20-item, four-factors scale, aims to measure the SDL ability among nursing students. The four factors are as follows: learning motivation (six items), planning and implementing (six items), self-monitoring (four items), and interpersonal communication (four items). 5-point Likert scale was used for rating each item, responses range from "Strongly Disagree" to "Strongly agree". Scores ranging from 20 to 100, with higher scores indicating higher SDL skills for nursing students. The Cronbach's α value of the original English scale was 0.916, and the Cronbach's α value four subscales ranged from 0.765 to 0.861.

## Procedures 

### Translation procedure

Researchers following the Brislin model to translate SDLI into Chinese [37].

- The first step: SDLI was independently translated into Chinese by two Chinese nursing professionals using the backward and forward translation procedure. Then, the first author compares and merges the Chinese scale translated by the two experts, and discusses the differences with the two experts until the final version is obtained.
- The second step: Two nursing professionals who were proficient in English and had no contact with the original scale independently performed reverse translation. The two post-translated scales were compared and the differences between the two translations were discussed in order to reach a consensus. The scale after translation is basically the same as the original scale.
- The third step: In this study, three researchers, including a psychologist and two nursing specialists, formed an expert panel. The panel compared the translation and back versions with the original scale, taking into account cultural adaptation and conceptual equivalence of idioms to make the language expressions more in line with the continental language conventions. Finally, 32 nursing students were selected through convenience sampling from Jinzhou Medical University for the preliminary experiment. Participants were asked to complete the scale. And the nursing students all expressed that the scale was thematically clear, structurally complete, logically coherent, and had no difficulty in semantic understanding.

### Data collection

The multistage sampling design was used to conduct this study. We randomly selected Jinzhou Medical University and Dalian University among 6 nursing colleges in Liaoning Province. Then 50% of classes are randomly selected from higher vocational colleges, the third batch of undergraduate students and the second batch of undergraduate students of the two selected universities, that is, 1–3 classes are selected in each grade, and 25–30 students are selected in each class. Results add up to1125 nursing students from 39 classes were selected. Jinzhou Medical University has 24 classes with 694 nursing students, Dalian University has 15 classes with 431 nursing students. The investigator explained the content, purpose and meaning of the questionnaire to the students before distribution, and reminded them to fill out the questionnaires carefully. After obtaining informed consent from the students, the questionnaires were distributed to the students on site and they completed the questionnaires independently. Nursing students completed the questionnaires in an average of 3.5 min and returned them on the spot after completion. Finally, 1087 out of 1125 nursing students participated in the questionnaire, and 975 students effectively completed the questionnaire. They included 636 students from Jinzhou Medical University and 339 students from Dalian University.

### Statistical analyses

SPSS (V26.0) and AMOS (V26.0) were used for statistical data analysis in this study. Demographic characteristics were analyzed using descriptive statistics (mean and standard deviation for continuous data and frequency and percentage for demographic characteristics).

### Items analysis

The total score of the translated scale was ranked from high to poor, and the relationship between the first 27% (high-score group) and the last 27% (poor-score group) was analyzed to judge whether the translated scale has an ideal discrimination ability. The correlation coefficients between the score of each item and the total score of the translated scale were calculated, as well as the Cronbach’ α value after the deletion of items. If Cronbach 'α value becomes larger after deleting the item, it can be considered to delete the item.

### Validity analysis

S-CVI > 0.9 and I-CVI > 0.78 indicated good content validity of the scale. In this study, seven experts were invited to evaluate the content validity of the translated scale using the Delphi method. The exploratory factor analysis (EFA) and confirmatory factor analysis (CFA) were performed to evaluate the underlying factor structure of the translated scale. And the sample of 975 cases was randomly subdivided into two groups, one (*n* = 509) for EFA and the other (*n* = 466) for CFA. Principal component analysis and orthogonal rotation variance maximization were used for exploratory factor analysis. AMOS was used for confirmatory factor analysis to analyze whether the fitting index of the model was appropriate.

### Reliability analysis

Cronbach 'α value and split reliability were used to evaluate the internal consistency of the scale. Two weeks after the first questionnaire, 60 nursing students were re-surveyed on SDL ability using the translated scale. The correlation between the results of the two surveys was analyzed to test the scale retest reliability and evaluate the consistency and stability of the scale throughout the data collection period.

### Ethics committee

All individuals have provided informed consent before the data collection.

## Results

### The sample

In this study, 975 nursing students in total were enrolled, including 109 males (11.2%) aged 18–25. And there were 866 female students (88.8%), aged from 17 to 26. The overall mean age was 20.55 ± 1.450. There are 289 students in grade one (29.6%), 412 students in grade two (42.3%) and 274 students in grade three (28.1%). Table 1 shows the demographic information of participants in this study. Table 2 shows the average SDLI score, The total mean was 48.178, SD = 9.20 and the means of each dimension were 14.1779, SD = 3.14; 12.4508, SD = 2.87; 12.3313, SD = 2.76 and 9.2182, SD = 2.25.

**Table1:** Frequency distribution of demographic characteristics(*n* = 975)

**Variables**	Groups	N	**%**
**City**	Jinzhou	636	65.23
	Dalian	339	34.77
**Sex**	Male	109	11.18
	Female	866	88.82
**Age(years)**	17	4	0.41
	18	57	5.82
	19	166	17.20
	20	283	29.33
	21	208	21.55
	22	164	16.99
	23	54	5.60
	24	22	2.28
	25	6	0.62
	26	1	0.10
**Grade**	Freshman	289	29.64
	Sophomore	412	42.26
	Junior	274	28.10
**Only child**	Yes	411	42.34
	No	562	57.76
**Education**	Higher vocational schools	298	30.56
	The third batch of undergraduate	182	18.67
	The second batch of undergraduate	495	50.77
**place of residence**	Urban	419	43.29
	Rural	452	46.69
	Suburban	97	10.02

**Table 2:** Mean (SD) scores and skewness and kurtosis values of the scale (*n* = 975)

Item	Mean ± SD	Skewness	Kurtosis
**learning motivation**	14.1779 ± 3.14	-0.034	0.698
**Q1**	2.346 ± 0.69	0.008	0.168
**Q2**	2.72 ± 0.78	0.03	0.246
**Q3**	2.358 ± 0.73	-0.187	-0.303
**Q4**	2.243 ± 0.74	0.212	0.066
**Q5**	2.288 ± 0.70	-0.093	-0.336
**Q6**	2.223 ± 0.70	0.294	0.357
**planning and** **implementing**	12.4508 ± 2.87	-0.017	1.004
**Q7**	2.482 ± 0.76	-0.124	-0.054
**Q8**	2.394 ± 0.77	0.114	0.012
**Q9**	2.225 ± 0.73	0.305	0.261
**Q10**	2.683 ± 0.75	-0.016	0.318
**Q11**	2.668 ± 0.76	-0.146	0.312
**self-monitoring**	12.3313 ± 2.76	-0.079	0.651
**Q12**	2.532 ± 0.74	0.069	0.163
**Q13**	2.497 ± 0.75	-0.086	-0.152
**Q14**	2.218 ± 0.72	0.337	0.226
**Q15**	2.634 ± 0.74	-0.014	0.224
**Q16**	2.45 ± 0.73	0.143	0.182
**interpersonal communication**	9.2182 ± 2.25	0.006	0.428
**Q17**	2.288 ± 0.78	0.2	-0.093
**Q18**	2.299 ± 0.78	0.172	-0.176
**Q19**	2.369 ± 0.74	-0.034	-0.288
**Q20**	2.262 ± 0.71	0.119	-0.033

### Item analysis

In the study, there was statistically significant (*P* < 0.001) in item-total score correlations based on Pearson correlation analysis, and correlations ranged from 0.533 to 0.708 (Table 3). In this study, the critical ratio of 20 items were 16.871 to 26.688 (Table 4). It shows that the items of the scale have good discrimination. Each item of the scale is positively correlated with the total score and moderately correlated with the scale. After deleting each item, Cronbach’ α value of the scale was 0.909 to 0.914, which does not exceed Cronbach’s α value of the scale (0.916).

**Table 3:** SDLI item-total score person correlation analysis results(*n* = 975, α = 0.05)

Item	Item content	*r*	*P*
**Q1**	I know what I need to learn	0.593	< 0.001
**Q2**	Regardless of the results or effectiveness of my learning, I still like learning	0.563	< 0.001
**Q3**	I strongly hope to constantly improve and excel in my learning	0.587	< 0.001
**Q4**	My successes and failures inspire me to continue learning	0.656	< 0.001
**Q5**	I enjoy finding answers to questions	0.596	< 0.001
**Q6**	I will not give up learning because I face some diffificulties	0.641	< 0.001
**Q7**	I can pro-actively establish my learning goals	0.644	< 0.001
**Q8**	I know what learning strategies are appropriate for me in reaching my learning goals	0.665	< 0.001
**Q9**	I set the priorities of my learning	0.661	< 0.001
**Q10**	Whether in the clinical practicum, classroom or on my own, I am able to follow my own plan of learning	0.633	< 0.001
**Q11**	I am good at arranging and controlling my learning time	0.672	< 0.001
**Q12**	I know how to find resources for my learning	0.644	< 0.001
**Q13**	I can connect new knowledge with my own personal experiences	0.637	< 0.001
**Q14**	I understand the strengths and weakness of my learning	0.623	< 0.001
**Q15**	I can monitor my learning progress	0.708	< 0.001
**Q16**	I can evaluate on my own my learning outcomes	0.666	< 0.001
**Q17**	My interaction with others helps me plan for further learning	0.542	< 0.001
**Q18**	I would like to learn the language and culture of those whom I frequently interact with	0.533	< 0.001
**Q19**	I am able to express messages effectively in oral presentations	0.598	< 0.001
**Q20**	I am able to communicate messages effectively in writing	0.556	< 0.001

**Table 4:** Discriminant validity analysis of the Chinese version of the scale (*n* = 975)

**Item**	**Low-score group** **mean** ± ***SD***	**High-score group** **mean** ± ***SD***	***t***	***P***
**Q1**	1.84 ± 0.62	2.81 ± 0.58	19.076	< 0.001
**Q2**	2.17 ± 0.73	3.19 ± 0.67	17.150	< 0.001
**Q3**	1.79 ± 0.63	2.82 ± 0.62	19.436	< 0.001
**Q4**	1.67 ± 0.57	2.80 ± 0.66	21.370	< 0.001
**Q5**	1.77 ± 0.59	2.75 ± 0.61	19.320	< 0.001
**Q6**	1.70 ± 0.54	2.76 ± 0.62	21.512	< 0.001
**Q7**	1.90 ± 0.70	3.00 ± 0.62	19.547	< 0.001
**Q8**	1.76 ± 0.60	2.97 ± 0.66	22.442	< 0.001
**Q9**	1.70 ± 0.61	2.79 ± 0.67	19.899	< 0.001
**Q10**	2.10 ± 0.69	3.16 ± 0.60	19.342	< 0.001
**Q11**	2.00 ± 0.64	3.19 ± 0.56	23.459	< 0.001
**Q12**	1.94 ± 0.63	3.04 ± 0.58	21.371	< 0.001
**Q13**	1.90 ± 0.64	3.00 ± 0.60	20.861	< 0.001
**Q14**	1.72 ± 0.58	2.77 ± 0.69	19.370	< 0.001
**Q15**	1.95 ± 0.57	3.20 ± 0.53	26.688	< 0.001
**Q16**	1.86 ± 0.60	3.01 ± 0.57	23.196	< 0.001
**Q17**	1.80 ± 0.69	2.78 ± 0.67	16.893	< 0.001
**Q18**	1.77 ± 0.65	2.79 ± 0.68	17.910	< 0.001
**Q19**	1.86 ± 0.62	2.89 ± 0.62	19.726	< 0.001
**Q20**	1.84 ± 0.63	2.75 ± 0.64	16.871	< 0.001

## Validity analysis

### Content validity

The Chinese version of SDLI was evaluated by a panel of seven experts. The evaluation results showed that the score range of the I-CVI was 0.86 -1.00, and the S-CVI value was 0.95.

## Construct validity

### Exploratory factor analysis

The Kaiser–Meyer–Olkin (KMO) test was 0.933, and Bartlett sphericity test was significant (χ2 = 3685.440; *P* < 0.001). Four factors supported by gravel maps accounted for 55.481% of the variance, respectively explaining 14.639%, 14.439%, 14.183% and 12.156%.

The Chinese version of SDLI is adjusted as follows. We adjusted item 12 (I know how to find resources for my learning.) from the planning and implementation dimension to the self-monitoring dimension. Finally, factor 1 includes six questions (Q1, Q2, Q3, Q4, Q5 and Q6), which are identified as "learning motivation". Factor 2 includes five questions (Q7, Q8, Q9, Q10 and Q11) identified as "planning and implementation"; Factor 3 includes five questions (Q12, Q13, Q14, Q15 and Q16) that are identified as "self-monitoring"; Factor 4 includes four questions (Q17, Q18, Q19 and Q20) identified as "interpersonal communication". The result is that each item has a higher load value than 0.40 on one of the common factors and there is no double-load phenomenon [38] (Table 5).

**Table 5:** Factor load and communalities of each item in SDLI of 20 Items(*n* = 509)

Item	F2	F3	F1	F4	Communalities
D11	0.741	-	-	-	0.664
D10	0.696	-	-	-	0.591
D7	0.656	-	-	-	0.607
D9	0.650	-	-	-	0.588
D8	0.516	-	-	-	0.484
D14	-	0.670	-	-	0.540
D16	-	0.664	-	-	0.586
D12	-	0.646	-	-	0.556
D13	-	0.579	-	-	0.488
D15	-	0.527	-	-	0.533
D3	-	-	0.748	-	0.632
D2	-	-	0.696	-	0.540
D4	-	-	0.673	-	0.615
D6	-	-	0.608	-	0.520
D5	-	-	0.491	-	0.401
D1	-	-	0.482	-	0.484
D18	-	-	-	0.762	0.635
D17	-	-	-	0.672	0.568
D19	-	-	-	0.628	0.567
D20	-	-	-	0.547	0.486

F1 contained Q1, Q2, Q3, Q4,Q5 and Q6, F2 contained Q7, Q8, Q9, Q10 and Q11, F3 contained Q12, Q13,Q14,Q15 and Q16, F4 contained Q17, Q18, Q19, and Q20

### Confirmatory factor analysis

**Fig.1:**
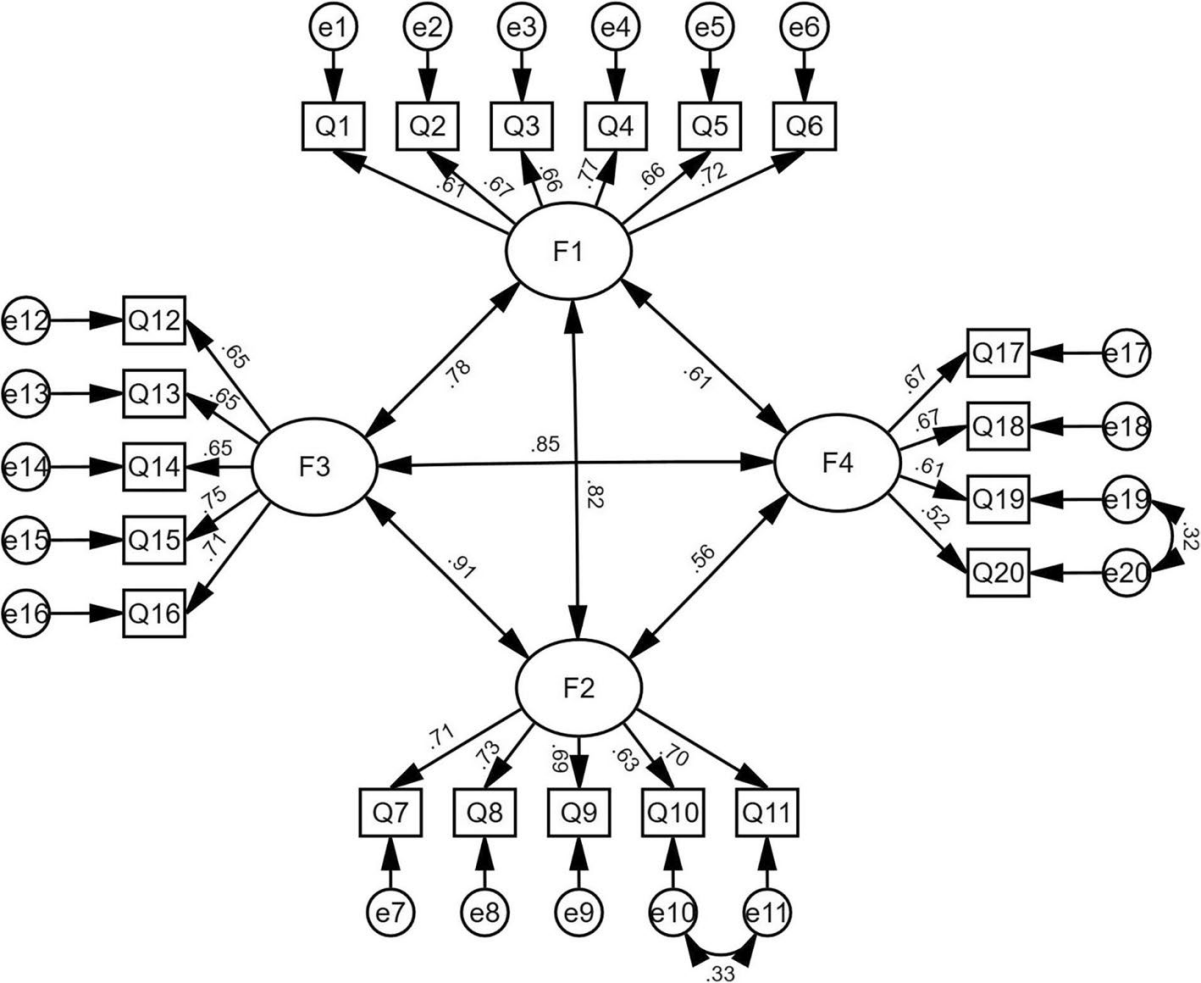
Standardized four-factor structural model of the Chinese version of the SDLI (*n* = 466)

The results of CFA are shown in Table 6. According to the modification indices (MI), the initial model was revised 2 times in order: e10 and e11; e19 and e20, respectively. In the final model fitness index (original model fitness index), the chi-square degree of freedom (CMIN/DF) was 2.285 (2.731), the comparative fit index (CFI) was 0.947 (0.928), and the incremental fit index (IFI) was 0.948 (0.929), and the tucker lewis index (TLI) was 0.938 (0.917) (Fig. 1).

**Table 6:** Evaluation fitness of SDLI model

Model	CMIN/DF	NFI	GFI	IFI	TLI	CFI	PNFI	PCFI
Initial model	2.731	0.892	0.908	0.929	0.917	0.928	0.770	0.801
Modified model	2.285	0.911	0.925	0.948	0.938	0.947	0.777	0.808
Standard value	< 5.000	> 0.900	> 0.900	> 0.900	> 0.900	> 0.900	> 0.500	> 0.500

### Reliability analysis

The Cronbach's α value of the Chinese version of SDLI was 0.916, and the Cronbach’s α values of the four factors ranged from 0.732 to 0.821.Two weeks after the first questionnaire, 60 nursing students were re-surveyed on SDL using the Chinese version of the SDLI with a retest reliability of 0.884.

## Discussion

The test results show that the Chinese version of SDLI has good psychometric characteristics and is a valid and reliable instrument. Hence, this scale can be used to measure the SDL ability of nursing students in China. According to the Chinese guidelines and common expressions, the obtained Chinese version of SDLI is appropriately different from the original scale. According to Brislin translation Principles [37], this study invited several nursing experts to adjust and translate the original scale according to Chinese expression habits and relevant guidelines, and finally formed the Chinese version of SDLI. There was good equivalence between the translated scale and the original scale. In this study, four factors were extracted, which were the same as the original scale, but some items were adjusted. Item 12 (I know how to find resources for my learning.) was adjusted from the second factor to the third factor. Because of this semantics, more emphasis is placed on self-management. The adjusted scale is more consistent with the Chinese cultural background. According to the survey, the translated scale was easy to understand and clear in structure. This scale was proved to be suitable for evaluating SDL ability of nursing students in Mainland China. And the score of each item was positively correlated with the total score of the scale.

In this study, Cronbach's α value and retest reliability were used to assess the scale's internal consistency and stability over time. The Cronbach’s α values of the total score was 0.916 and the Cronbach’s α values of four factors ranged from 0.732 to 0.821. The results showed that there was internal correlation or good homogeneity among the 20 items. The retest reliability of the scale was 0.884, indicating high stability over time and repeatability in nursing students. Overall, the Chinese version of SDLI is reasonably reliable among nursing students.

This study evaluated the structural validity and content validity of the Chinese version of SDLI. ICVI was 0.86—1.00 and S-CVI was 0.95, showing good content validity. The ideal result is that each item has a load value greater than 0.40 on a common factor, and there is no double-load phenomenon, and the cumulative variance contribution rate of extracted common factors is not less than 40% [38]. The final model of the Taiwan version of SDLI test, using maximum likelihood estimation, had factor loadings greater than 0.40 for 19 items, except for one item (0.39). In the Chinese version of SDLI, four factors could explain 55.418% of the variation. And the Communalities of each item ranged from 0.401 to 0.664, indicating good structural validity.

In this study, the measured values of the model fit well, CMIN/DF = 2.285, CFI = 0.947, IFI = 0.948, TLI = 0.938. The results show that the model has strong factor loadings and explanatory differences. CFA results confirmed that the Chinese version of SDLI had a better fitting index. In terms of discriminant validity, the scores of high and low groups were significant (*P* < 0.001). Overall, the Chinese version of SDLI has appropriate validity among nursing students. The correlation between the total scores was statistically significant, with the range of 0.533 and 0.708, within the recommended standard range (not less than 0.4).

The results show that the Chinese version of SDLI has good homogeneity, stability, structure validity, content validity and discriminant validity. Therefore, the Chinese version of SDLI is an appropriate tool to evaluate the SDL ability among nursing students.

## Limitations

We only conducted a cross-sectional study in our study, and further longitudinal studies are needed to determine the results. The study focused on nursing students in two Chinese cities.The adaptability of the Chinese version of SDLI to other parts of China should also be further verified.

## Conclusion

This study investigated the psychometric properties of the Chinese version of SDLI, and the results showed that the scale had good validity and reliability. The scale is simple in content and structure and flexible in evaluation method, which can be used to measure SDL ability of nursing students. Under the background of the rapid development of nursing discipline and the constant change of medical environment, this provides an effective measurement tool to improve the SDL ability of Chinese nursing students. It also provides a basis for educators to cultivate students' ability to actively acquire knowledge and lifelong learning through SDL.

## Supplementary Information

**Additional file 1.**

## Data Availability

The datasets generated and/or analyzed during the current study are not publicly available to preserve anonymity of the respondents but are available from the corresponding author on reasonable request.

## Abbreviations

SDLI: Self-directed learning instrument
SDL: Self-directed learning
CMIN/DF: chi-square/degree of freedom
CFI: Comparative fit index
TLI: Tucker lewis index
IFI: Incremental fit index
SDLRS: Self-directed learning readiness scale
SRSSDL: Self Rating Scale of Self Directed Learning
SDLRSNE: Self-Directed learning readiness scale for nursing education
S-CVI: Scale-level content validity index
I-CVI: Item-level content validity index
KMO: The Kaiser–Meyer–Olkin
CFA: Confirmatory factor analysis
MI: The modification indices
CR: Critical ration
