# Supplementary material for: Reliability and validity of the Chinese version of the self-directed learning instrument in Chinese nursing students
Source: BMC Nurs. 2023 Feb 24;22:51. doi: 10.1186/s12912-023-01201-3 (PMC9950017; doi:10.1186/s12912-023-01201-3)
Supplement: Supplementary file 1 — Additional file 1. [file 12912_2023_1201_MOESM1_ESM.docx]

**Self-Directed Learning Instrument (SDLI) for Nursing Students (English version and Chinese version)**

| **Item** | **Item content (English/Chinese)** | **Score** |
| --- | --- | --- |
| Q1 | I know what I need to learn. | 1 2 3 4 5 |
|  | 我知道自己需要学什么？ |  |
| Q2 | Regardless of the results or effectiveness of my learning, I still like learning. | 1 2 3 4 5 |
|  | 不论学习成效如何，我仍然喜欢学习？ |  |
| Q3 | I strongly hope to constantly improve and excel in my learning. | 1 2 3 4 5 |
|  | 我渴望在学习上精益求精？ |  |
| Q4 | My successes and failures inspire me to continue learning. | 1 2 3 4 5 |
|  | 我的成功或失败经验都会激励我持续去学习？ |  |
| Q5 | I enjoy finding answers to questions. | 1 2 3 4 5 |
|  | 我乐于寻找问题的答案？ |  |
| Q6 | I will not give up learning because I face some diffificulties. | 1 2 3 4 5 |
|  | 我不会因为学习遇到困难而放弃学习？ |  |
| Q7 | I can pro-actively establish my learning goals. | 1 2 3 4 5 |
|  | 我能主动制定自己的学习目标？ |  |
| Q8 | I know what learning strategies are appropriate for me in reaching my learning goals. | 1 2 3 4 5 |
|  | 我知道我适合用什么学习方法来达到自己期望的目标？ |  |
| Q9 | I set the priorities of my learning. | 1 2 3 4 5 |
|  | 我会设定学习的优先顺序？ |  |
| Q10 | Whether in the clinical practicum, classroom or on my own, I am able to follow my own plan of learning.  不论是上课、实习或自修，我都能依据自己的计划来学习？ | 1 2 3 4 5 |
| Q11 | I am good at arranging and controlling my learning time. | 1 2 3 4 5 |
|  | 我善于安排与控制学习的时间？ |  |
| Q12 | I know how to find resources for my learning.  我知道如何寻找资源来学习？ | 1 2 3 4 5 |
| Q13 | I can connect new knowledge with my own personal experiences | 1 2 3 4 5 |
|  | 我能将新的知识与个人的经验做连接？ |  |
| Q14 | I understand the strengths and weakness of my learning. | 1 2 3 4 5 |
|  | 我了解自己在学习方面的优缺点？ |  |
| Q15 | I can monitor my learning progress. | 1 2 3 4 5 |
|  | 我能监测自己的学习进展？ |  |
| Q16 | I can evaluate on my own my learning outcomes. | 1 2 3 4 5 |
|  | 我能自我评价学习结果？ |  |
| Q17 | My interaction with others helps me plan for further learning. | 1 2 3 4 5 |
|  | 与他人的互动能帮助我规划进一步的学习？ |  |
| Q18 | I would like to learn the language and culture of those whom I frequently interact with. | 1 2 3 4 5 |
|  | 我会想要学习与我经常互动人的语言及文化？ |  |
| Q19 | I am able to express messages effectively in oral presentations. | 1 2 3 4 5 |
|  | 我能以口语的方式有效表达讯息？ |  |
| Q20 | I am able to communicate messages effectively in writing. | 1 2 3 4 5 |
|  | 我能以书写的方式有效传递讯息？ |  |
